# A personhood and citizenship training workshop for care home staff to potentially increase wellbeing of residents with dementia: intervention development and feasibility testing of a cluster randomised controlled trial

**DOI:** 10.1186/s40814-022-01222-w

**Published:** 2023-01-09

**Authors:** Jason Corner, Bridget Penhale, Antony Arthur

**Affiliations:** University of East Anglia, School of Health Sciences, Norwich Research Park, Norwich, NR4 7TJ UK

**Keywords:** Dementia, Care home, Personhood, Citizenship, Dementia care mapping, Training

## Abstract

**Background:**

In the UK, one third of people with dementia live in residential care homes, a sector where high staff turnover negatively affects continuity of care. To examine the effect of including personhood and citizenship principles in training, interventions need to be robustly tested, with outcomes relevant to residents with dementia.

**Methods:**

*Phase one intervention development*: The training intervention (PERSONABLE) comprised five reflective exercises facilitated by a mental health nurse/researcher. PERSONABLE was informed by four focus groups, and one field exercise, consisting of care home staff and family members. *Phase two feasibility testing*: Participants were (i) care home residents with dementia and (ii) care home staff working in any role. After baseline measurements, care homes were randomly allocated to (i) staff receiving PERSONABLE training or (ii) training as usual. Feasibility outcomes were the recruitment and attrition of care homes, residents and staff members (measured ten weeks between randomisation and follow-up), the acceptability of the training intervention PERSONABLE, and acceptability of outcome measures. The care home environment was evaluated, at baseline, using the Therapeutic Environment Screening Survey for Residential Care Homes. Measurements conducted at baseline and follow-up were resident wellbeing (Dementia Care Mapping™), staff knowledge of and confidence with personhood and citizenship (Personhood in Dementia Questionnaire and a perceived ability to care visual analogue scale). Inter-rater agreement for Dementia Care Mapping™ was undertaken at follow-up in one intervention and one training as a usual care home.

**Results:**

*Phase one*: The developed reflective approach to the PERSONABLE exercises appeared to give staff a holistic understanding of residents living with dementia, seeing them as autonomous people rather than reductively as persons with a condition. *Phase two:* Six care homes, 40 residents and 118 staff were recruited. Four residents were lost to follow-up. Twenty-nine staff in the PERSONABLE arm of the study received the training intervention. In the PERSONABLE arm, 26 staff completed both baseline and follow-up measurements compared to 21 in the training as the usual arm. The most common reason for the loss to follow-up of staff was leaving employment. For the outcome measure Dementia Care Mapping™, the proportion of overall agreement between the two observers was 18.6%. High attrition of staff occurred in those homes undergoing leadership changes.

**Conclusion:**

With the right approach, it is possible to achieve good engagement during trial recruitment and intervention delivery of care home managers, staff and residents. Organisational changes are a less controllable aspect of trials but having a visible researcher presence during data collection helps to capitalise the engagement of those staff remaining in employment. Tailored, brief and flexible training interventions encourage staff participation. Simplification of study methods helps promote and retain sufficient staff in a definitive randomised controlled trial. This study found that some components of Dementia Care Mapping™ work effectively as an outcome measure. However, inter-rater reliability was poor, and the practical implementation of the measurement would need a great deal of further refinement to accurately capture the effect of a training intervention if delivered across a large number of clusters. The Dementia Care Mapping™ measurement fidelity issue would be further complicated if using multiple different unacquainted observers.

**Trial registration:**

Registered with the ISRCTN under the title: Does a dementia workshop, delivered to residential care home staff, improve the wellbeing of residents with dementia? Trial identifier: ISRCTN13641553. Registered: 30/05/2017 http://www.isrctn.com/ISRCTN13641553.

## Background

*“I am a human being. I still exist … what I ask for is that what is left of my life shall have some meaning. Give me something to die for!”*

George Thomas (a person with dementia), in ([9], page 31).

George is one of an estimated 815,827 people with dementia living in the UK [34]. Around 291,000 people in England and Wales live in care homes, a diverse group needing individualised care (Office for National Statistics, [24, 34]). The identity and sense of purpose of this group of people can be compromised by limited access to the diverse community beyond the care home walls [23].

Care staff turnover is around a third in the first year of employment [10] inevitably affecting the continuity of care [21]. Increasing the provision of training has the potential to improve staff wellbeing and turnover [14, 25]. The Care Certificate, developed in response to The Cavendish Report [12], is usually delivered via eLearning, mostly covering basic practical skills needed for care, but does little to enhance staff understanding of person-centred care [15].

Conducting trials in care homes has presented many challenges [29], and in the EPIC trial, the ability to obtain sufficient data was reportedly limited by the demands of busy care environments and high staff turnover. One approach that indicates better success with intervention engagement is when a study takes a more bespoke approach such as in the WHELD study [2] [13]. This study reiterating the recommendations that research conducted in care homes be adapted to the practical demands of these busy work environments. Other authors [18] have highlighted the role that care home managers can play in the initial stages of a trial, when good engagement can positively affect trial recruitment of care homes.

Many researchers who have designed dementia training for interventions include a component of personhood but to a lesser extent citizenship principles [22]. Personhood is often used as a universal term, encompassing aspects of the community ‘lens’ of citizenship [4]). Attempts have been made to [33] differentiate the two theories as ‘seeing the person’ (personhood) and ‘seeing the person as an active social agent’ (citizenship). Despite the similarities between personhood and citizenship the two theories have a distinct utility when applied to residential care home settings. For the purposes of this intervention, the two concepts were specifically defined (Table 1).

**Table 1:** The key components of personhood and citizenship as defined in this study

Personhood	Citizenship
Identity focussed	Community focussed
Internal attributes	Societal attributes
Individualism	Communalism
Agency within self	Agency with others

### Study objectives

This feasibility study aimed to assess the acceptability and feasibility of conducting PERSONABLE, a short reflective personhood and citizenship workshop intervention, in residential care homes and piloting the intervention within a cluster randomised trial. Specific study objectives were as follows:

1. *Intervention development and acceptability*: to develop personhood and citizenship training among care home staff and evaluate the acceptability of the finalised PERSONABLE intervention.
2. *Participant recruitment and retention*: to estimate the flow of residents, staff, and care homes in a future definitive randomised controlled trial.
3. *Outcome measurement suitability*: assess the acceptability to residents and staff of outcome measures and their potential for detecting any possible effect of the PERSONABLE intervention.

## Methods

### Design

The study was delivered in two phases. *Phase one*: intervention development using focus groups. *Phase two*: testing the feasibility of an intervention within a cluster RCT methodology.

### Phase one: intervention development methods

To build on the original idea for the personhood and citizenship training intervention, a series of four focus groups were conducted in three residential care homes. Topic guides were adapted to reflect the potential diversity of participants while retaining the focus on intervention development. Sequentially gathering focus group data and then revising the PERSONABLE exercises iteratively informed intervention adaptations. Focus group dynamics and discourse were observed by a nurse, independent to the PERSONABLE study team and experienced in the needs of residential care homes. Focus group data were analysed using framework analysis (Smith and Firth, [28]). Mapping data by job role informed changes to PERSONABLE tailored to potentially diverse staff experiences and work practices. To highlight practical areas in need of consideration, the core issues arising from the framework analysis were separated into those which enhanced or detracted from a personhood or citizenship approach to care, or factors which might affect the effective delivery of a training intervention. The framework was then mapped against the topic guide themes of (1) staff characteristics, (2) current training, (3) personhood and citizenship, and (4) PERSONABLE feedback. Subsequent changes to the PERSONABLE exercises were made in conjunction with a working group of professionals familiar with dementia care and adaptations were carried into subsequent focus groups.

Once adaptations informed by focus group data were complete the PERSONABLE intervention was delivered to a group of care staff and subsequently further refinements were made. This fieldwork exercise was observed by AA, a nurse experienced in the care of people with dementia.

### Phase one: results

The four focus groups comprised a total of 12 care home staff, working in a variety of roles, and three family carers. The fieldwork, which followed the focus groups, had five care worker participants and occurred in a care home independent of the focus group sites. Following the focus groups and fieldwork, the exact design and delivery of PERSONABLE was finalised. Changes made during this process related to participant comments on (i) the practical delivery of an intervention, which considered length, time and days of the week, universally the staff groups asserted delivery of an intervention should be brief in the quiet afternoon period of the day; (ii) the mode of delivery, whether a taught or more reflective and applied approach should be undertaken, with all staff groups reporting a preference for more reflective approaches to their learning; and (iii) the content of the intervention, type of information and the level at which content should be pitched, interestingly in this domain there was no significant variation in the knowledge and understanding demonstrated by care worker staff, or those with less ‘care’related patient contact such as cleaning staff, each staff group having their own vocation related observations on resident experiences.

The final version of PERSONABLE consisted of five consecutive elements: (1) Exercise one ‘from waking to work’: resident choice and autonomy; (2) Exercise two ‘reflections on personhood’: reflecting on staff personhood; (3) Exercise three ‘reflections on personhood’: reflecting on resident personhood; (4) Exercise four ‘from outside to inside’: replicating community diversity; and (5) Exercise five ‘the pledge’: turning reflection into action. The choices made when adapting the original idea for the workshop into this finalised version are summarised in Table 2.

**Table 2:** Adaptations to PERSONABLE after analysing focus group data

**Waking to work exercise -** *Refining the key purpose to exploring the utility of resident ‘choice’.* Initially designed to address resident choice and community diversity. All groups commented that the exercise did not make them think about community diversity ‘it highlighted more about choice but not what you said about the community’ and ‘it’s more about choice than (the community)’. Staff readily understood the need for resident choice ‘loads of choices that we can make every day and take for granted’, but not necessarily as a platform for considering the underlying citizenship of being assisted to make autonomous decisions; therefore, this was added as a teaching point to the final version of PERSONABLE.	
**Exercises two and three: Personhood -** Following focus group feedback, the personhood domain relating to ‘neurological impairment’ changed to ‘How I learn’. Consensus from all groups that ‘neurological impairment’ implies disablement. All groups felt any replacement term should not be medical so that a person with limited experience might understand and utilise the term, when the focus group facilitator asked, ‘would you understand the term cognitive function?’, one participant replied, ‘a lay person wouldn’t’. One family member suggested ‘my learning style’, which led to discussion exploring the positive utility of viewing the person with dementia as having the capacity to learn ‘are you really trying to find out how they absorb information?’. There was consensus that ‘how I learn’ possessed a powerful mechanism to convey the principles of personhood and citizenship.	
**Exercises two and three: Personhood -** Replacement term for central circle of personhood model denoting ‘staff’ and ‘resident’. The terms ‘staff’ and ‘resident’ replaced with one term ‘who am I?’ for both exercises. Staff broadly expressed terms in exercises two and three should be consistent for staff and the resident. A participant in focus group two implied they perceived the assessment of personhood as the same regardless of whether the person has dementia or not ‘I probably looked at this and thought of myself before I thought of anyone with dementia’. A participant in group one also commented that ‘you shouldn’t really talk to people who have got dementia any different than somebody who hasn’t got dementia’. When reviewing these data, the research team concluded that the central circle should have the unifying phrase ‘who am I?’ for both staff and residents.	
**Outside to inside exercise -** Simplifying language to emphasise key concepts. After observing that participants spent much of the focus group reading text related to the original exercises, it was agreed that minimal text should be included on the revised PERSONABLE worksheets, with the aim of improving staff engagement with the reflective discussions.	
**The Pledge -** More detail added to the pledge instruction. After the focus groups concluded, the wording of the pledge was changed from the simple statement ‘For the next 30 days I will’ to ‘Within the next 30 days I will change one thing about the way I work that may improve my understanding of a resident who has dementia. Or I might introduce something from the outside community into the care home’. This decision was based on feedback in the focused discussion groups that some staff might have difficulty thinking of a pledge ‘if they can’t see it, just a few examples and they may come up with’ and ‘if there’s examples there, you can sort of say ‘I see where that’s coming from’ and maybe something new’. The text was adjusted to provide more guidance and some non-specific, and general, examples were provided.	

The PERSONABLE exercises aimed to give staff a holistic understanding of residents living with dementia and seeing them as autonomous people (exercise one) rather than reductively as persons with a condition. The staff considered their own personhood (exercise two) and were then asked to compare this personal appraisal with an appraisal of the personhood of a resident with dementia (exercise three). PERSONABLE was designed to help participants consider how they might promote a sense of community and purpose (exercise four) and encourage these care practices within the residential care environment (exercise five).

### Phase two: methods

#### Setting

The study took place in care homes in the East of England. A mixture of small and larger care homes located in urban and rural locations was approached.

#### Participants

##### Care home

Residential care homes in the East of England were eligible if they provided care for people with dementia. *Resident*: Residents were eligible if they had a diagnosis of dementia with either capacity to consent to participation in the study or who had a consultee as outlined within the terms of the Mental Capacity Act 2005 (Department of Health, [11]). Residents were excluded if they were receiving palliative care or had an acute illness. *Staff*: Staff was eligible if they were employed on a full or part-time basis, working in any role such as care worker, ancillary, maintenance or administrative positions. Senior management was excluded because of their potential involvement in resident participant selection.

#### Baseline measurements

All baseline measurements were collected by JC prior to randomisation in all six sites. Descriptive information about each care home was obtained from the care home manager, including care home ownership, number of resident bedrooms and number of staff.

##### Residential care home

The quality of the usual care environment was measured using the Therapeutic Environment Screening Survey for Residential Care (TESS-RC) [27], which consists of 31 items. In response to comments from the ethics committee granting permission to conduct this research, only the 23 items relating to public areas of the care home were used.

##### Dementia Care Mapping™

Dementia Care Mapping™ (DCM™) was originally developed [19, 20] as a practice development tool. Version 8 of the DCM™ observational measure was used for this study, and the measure has been reported as valid and reliable [6]. In DCM™, an observer attempts to interpret the experience of those who have dementia and then reflectively discuss the observation data with staff [7, 17, 31, 32]. Because of the inter-relatedness of data collection, reflective staff discussion, staff adaptions to care and further data collection, DCM™ has rarely been used, in trials, solely as an outcome measure. Even when trials have stated DCM™ was used as an outcome measure, the observational data remains intrinsically linked to the intervention of reflective discussion with staff [8].

DCM™ comprises three elements. For every resident participant, each of these three elements was recorded in 5-min intervals over a 4-h period at baseline and at follow-up:

1. *Mood and Engagement score*: refers to two concepts ‘mood’ and ‘engagement’ recorded as one value, the one with the most potential for resident wellbeing. For example, for a person presenting as very engaged in a leisure activity but observed to have a neutral mood, the value for the engagement would be recorded. Often trials use a DCM™ wellbeing/illbeing ‘WIB’ score as the primary outcome measure, which is the mean of the aggregate mood and engagement score for each participant.
2. *Behaviour Category Code*: there are twenty-three different behaviour category codes designed to intricately describe the behaviours of people with dementia. Learning the hierarchy of DCM™ coding of behaviours is somewhat complex, in most cases the most prevalent behaviour with the greatest potential for wellbeing is recorded. For example, if a person is observed to be talking (Articulation) whilst walking (Kum and go) in equal measure during the 5-min time frame, the one with the most potential for wellbeing should be recorded.
3. *Personal Enhancing or Personal Detracting*: DCM™ attempts to describe the nature of interactions which occur between the staff and those they care for, using thirty-four codes split into detracting or enhancing. The DCM™ manual states that interactions which are significant should be recorded, for this study all interactions were recorded, for example, even brief fleeting interactions such as nods, or one-word ‘hello’ greetings.

#### Residential care home staff

Residential care home staff knowledge about personhood was measured using the Personhood in Dementia Questionnaire (PDQ), a tool created and reported as valid and reliable by [16]. The tool consists of twenty statements that a member of staff can rate, on a seven-point Likert scale, between agree strongly (7) and disagree strongly (1). How able the staff felt to deal with residents who had dementia was measured using a zero to 100 visual analogue scale (VAS). The VAS scale format has been suggested as an accessible way for people to inductively measure a given phenomenon [1].

#### Randomisation

Cluster randomisation occurred at the level of the care home. Block randomisation was used with a block size of two. Once two sites had been recruited and baseline measures complete, they were randomised by AA, using the ralloc command in Stata version 14, on a 1:1 basis (PERSONABLE intervention or training as usual).

#### Intervention

The PERSONABLE dementia workshop was facilitated face-to-face in a private room by JC across all intervention sites. In response to phase one focus group data, PERSONABLE was designed to be brief, lasting no longer than 1 h and fitting into the potentially quiet period after staff handover. The workshop comprised of five reflective exercises, described in phase one results below. PERSONABLE required minimal resources other than a simple and predominantly illustrative workbook which assisted the facilitator when guiding staff participants through the PERSONABLE workshop. Staff participants in the intervention arm were offered the PERSONABLE intervention once per participant. This was a waitlist trial and those randomised to the training as the usual group were offered PERSONABLE once all follow-up data had been collected.

### Training as usual

The control group received training as usual. During phase one, focus group key data was collected about the usual type, content, length and frequency of training as usual. These data were collected for staff working across multiple roles and indicated that residential care homes typically provide (i) mandatory training with limited dementia-specific content; (ii) occasional dementia-specific training usually attended by senior staff and subsequently informally disseminated to junior care staff; (iii) usually no dementia-specific training for ancillary, maintenance and administration staff; and (iv) when dementia training is delivered, it does not usually explore principles relating to personhood or citizenship.

### Follow-up measurements

All follow-up measurements were assessed ten weeks post-randomisation to allow time for intervention delivery and for the staff to complete their pledges, a component of the intervention. All measurements taken at baseline, at the level of staff and residents, were repeated. DCM™ inter-rater agreement was assessed at follow-up by JC, and a DCM™ mapper independent of the study, at one intervention and one training as the usual site. The second rater was experienced in the application of DCM™ and involved in the training of DCM™ practitioners. The observers took regular breaks to discuss the observation and refer to the DCM™ handbook [30]. To ensure a true representation of inter-rater agreement, even if a consensus was reached after discussion, the initial observation data was not altered.

### Sample size

As the study was not a definitive randomised controlled trial the aim of the analysis was not to determine evidence of effect and therefore not powered as such. To help ensure the smooth running of trial procedures, recruitment was limited to forty residents across the six care home sites, and this number was derived from a 4-h scoping exercise of DCM™ conducted in a care home separate from phase two. There was no limit on staff recruitment within the participating homes. Care home recruitment was limited to six sites to help ensure meaningful feasibility data could be collected.

#### Blinding

The person responsible for randomisation (AA) was blind to care home identity but blinding of allocation was not possible for JC or care home managers and staff. At follow-up, attempts were made to blind the second person undertaking DCM™ to study arm allocation. Staff and resident participants were assigned a code and JC remained unaware of which code represented which study arm during analysis.

### Analysis

DCM™ mood and engagement are measured on a −5 to +5 ordinal scale. DCM™ denotes a neutral state of mood or engagement with a +1 score. The frequency of the 23 behaviour category codes in each trial arm was explored by grouping the individual codes by their potential (high, moderate, low or none) for mood or engagement, as described in the DCM™ handbook [30]. The no potential for wellbeing behaviour code is ‘N’ when a participant is asleep, or ‘Land of Nod’. The use of personal enhancers and detractors is descriptive of the nature of the staff interaction rather than a numerical measure of interaction quality. There are seventeen personal enhancing staff interaction categories and seventeen detracting staff interaction categories. Usually, these codes are only used when an interaction of interest has taken place. However, for this study to capture the breadth of interactions across all sites, all interactions occurring between staff and residents were recorded. Because of the high number of interactions anticipated from taking this approach the rating of the thirty-four different interactions was condensed to enhancing (+1) and detracting (−1). DCM™ data can be converted into a concordance coefficient to describe interrater agreement. The concordance coefficient is the percentage agreement between different observers for the behaviour category codes and mood and engagement scores, across the 5-min time frames for which both observers have data.

The PDQ uses a seven-point scale for each of the 20 items. This scale was adjusted to 0–6 to include a zero value. The VAS and PDQ data were reported as a mean for each group and data captured for missing cases and outcome concordance.

Study data was analysed using the Statistical Package for the Social Sciences (version 25). Analysis was predominantly descriptive in accordance with the feasibility design and the study objectives. Staff flow through the study was mapped at baseline, intervention delivery and follow-up to inform decisions on how future trials might weigh recruitment.

### Phase two: results

#### Recruitment and participant flow

##### Care home

Six care homes agreed to take part in the study. Twenty-seven care homes from a range of settings and sizes were approached for inclusion in the study. The most frequent reason for non-participation of care homes was a perceived time burden of research participation by managers. This initial reluctance was somewhat reduced when managers became aware of the PERSONABLE intervention, recognising the training as potentially beneficial. Care homes tended to decline participation if already participating in another study or if study timings coincided with a Care Quality Commission inspection. All recruited care homes remained in the study for baseline and follow-up measurements, even when one care home in the PERSONABLE arm lost both the deputy and general manager between baseline and follow-up measurements.

##### Staff

**Fig 1:**
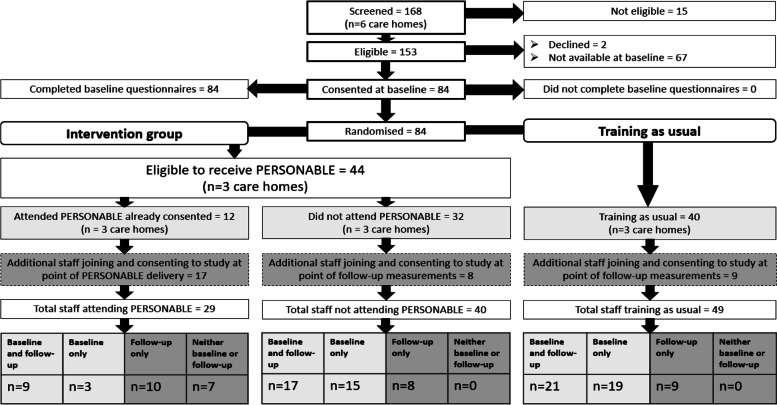
Study flow: residential care home staff

Of the 168 staff screened for eligibility for the study, 154 met the eligibility criteria (Fig. 1); 14 were working in management positions and therefore excluded. Most of the staff lost to follow-up were those who had left employment, 13 of whom were from care homes in the PERSONABLE arm; 11 of these 13 were from the same home, where between baseline and follow-up there was a change in both the general and deputy care home managers. In the training as usual arm 10 staff were lost to follow-up due to leaving employment, a large proportion of these (*n*=6) were from a small care home (*n*=11 total staff) undergoing a change of management. There was a balance between trial arms for staff who were away from work, on holiday or who had prolonged gaps in shifts and therefore missed follow-up measurements. Only two participants, both in the training as usual arm, declined to complete follow-up questionnaires when they had already completed baseline questionnaires.

##### Residents

**Fig. 2:**
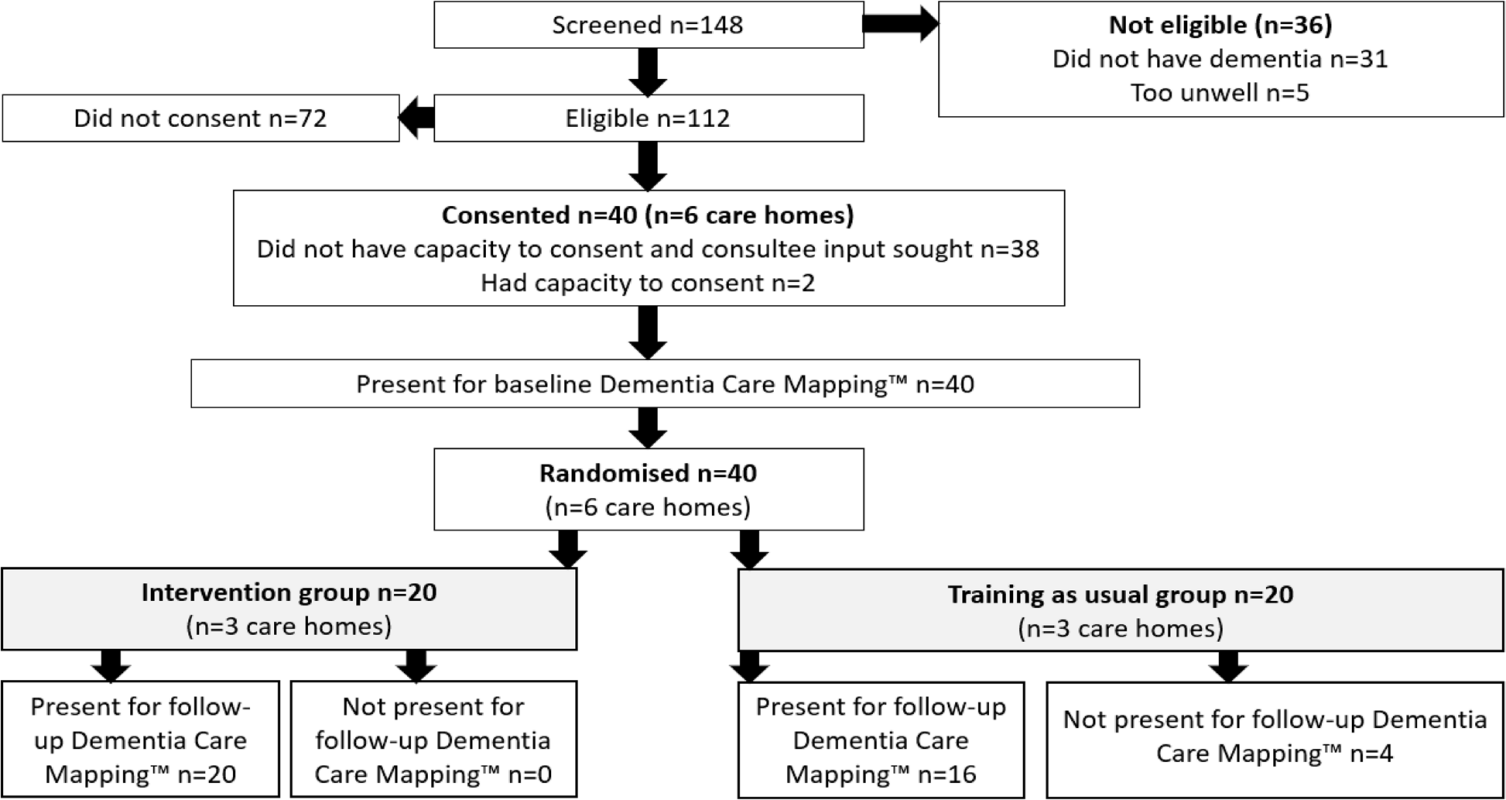
Study flow: recruitment and retention of resident participants

Of the 148 residents screened for eligibility for the study, 112 met the eligibility criteria (Fig. 2). Before being approached for inclusion, the ability of eligible residents to provide meaningful consent was discussed with the care home manager. Only two of the 40 recruited residents were assessed as having the capacity to consent to participation in the study. The other 38 residents were recruited following the use of the consultee process. In the training as usual arm, one gentleman was initially recruited but no data were collected. They were originally reported to have dementia, but it soon transpired his diagnosis had been incorrectly reported.

Four residents were not available at follow-up; one resident in the PERSONABLE arm was receiving palliative care and three residents in one care home in the training as usual arm had moved to different care homes. At follow-up, a resident in a PERSONABLE arm care home was recovering from a hip operation but remained included in the study because she was being inclusively rehabilitated within the communal areas of the care home.

##### Groups at baseline

Despite the small number of clusters, baseline characteristics for many variables were balanced between both arms of the trial (Table 3). Care home characteristics of trial arms were mostly equivalent, except for the mean TESS-RC scores, 49.3 for PERSONABLE and 38.7 for training as usual homes, indicating a greater level of environmental care quality in the intervention arm. The ratio of female to male residents differed between trial arms, with 80% male and 20% female residents in the PERSONABLE arm and 45% male and 55% female participants in the training as the usual arm. This was most likely due to one care home with an unusually high proportion of male residents being randomised into the PERSONABLE arm. Staff characteristics were similar for gender and work role between both arms. Although behaviour category codes for high-potential behaviours were balanced between trial arms, there were a higher number of medium potential behaviours observed in the PERSONABLE (286) than the training as usual (179) arm and a much higher incidence of behaviours with no potential for wellbeing (sleep) in the training as usual arm (152) compared to the PERSONABLE arm (46).

**Table 3:** Groups at baseline

Level of data				PERSONABLE	Training as usual
Care home (*n*=6)	Total whole-time equivalent staff		Mean (range)	26.3 (13–43)	25.0 (13–34)
	Total bedrooms		Mean (range)	26.0 (12–51)	22.7 (16–31)
	Rural		*n*	2	2
	Urban		*n*	1	1
	Therapeutic Environment Screening Survey		Mean (range)	49.3 (33–58)	38.7 (37–42)
Resident (*n*=40)	Gender		Female, *n* (%)	16 (80.0)	11 (55.0)
			Male, *n* (%)	4 (20.0)	9 (45.0)
		Missing cases	N		1
Observation (*n*=1584)^a^	Wellbeing/illbeing score		Mean (SD)	1.1 (0.2)	1.2 (0.5)
	Personal detractors		*n* (%)	3 (1.8)	2 (1.3)
	Personal enhancers		*n* (%)	165 (98.2)	152 (98.7)
	High-potential behaviours		*n* (%)	454 (54.6)	445 (56.6)
	Medium potential behaviours		*n* (%)	286 (34.4)	179 (22.8)
	Low-potential behaviours		*n* (%)	46 (5.5)	10 (1.3)
	No potential behaviours		*n* (%)	46 (5.5)	152 (19.3)
Staff (*n*=118)	Gender	Female	*n* (%)	62 (90.5)	44 (89.8)
		Male	*n* (%)	6 (9.5)	5 (10.2)
		Missing cases	*N*	1	
	Months experience		Mean % (SD)	29.4 (13.3)	24.9 (8.8)
	Staff role	Care worker	*n* (%)	52.0 (75.4)	36.0 (74.9)
		Ancillary	*n* (%)	11.0 (15.9)	8.0 (17.6)
		Other	*n* (%)	6.0 (8.7)	3.0 (7.5)
		Missing cases	*N*		2
	Personhood in Dementia Questionnaire (0–100)		Mean (SD)	89.5 (10.8)	86.6 (11.3)
	Visual Analogue Scale (0–100)		Mean score (SD)	85.0 (18.7)	83.3 (16.2)

^a^Five-minute resident observation intervals for which data was available, out of a total of 1920 possible five-minute observation intervals

#### Intervention delivery

PERSONABLE was delivered successfully at the three sites randomly allocated to receive the training intervention. All care homes requested that PERSONABLE be delivered during the staff handover period just after lunch. Despite the presence of a training intervention attracting interest during recruitment, because of organisational pressures, none of the intervention care homes felt able to facilitate more than one session of PERSONABLE. One care manager understandably rearranged the date of PERSONABLE because of a resident emergency. Despite these challenges, of the 69 recruited staff in the intervention arm, 29 attended PERSONABLE. Across the three intervention sites, care workers (*n*=20) were most predominant in attendance. However, in one care home, there were more ancillary staff (*n*=7) who attended PERSONABLE than care workers (*n*=4).

The content of the PERSONABLE intervention was well received; all staff roles were willing to engage in reflective conversations about their work. The pledges created by staff were thoughtful and largely reflective of an understanding of either personhood or citizenship theory, twelve pledges were reflective of personhood theory, such as ‘try to chat more about things they have done during their life and learn more about them’. Thirteen pledges were reflective of citizenship theory ‘bring in my niece and pets to visit more often to cheer up residents’. Pledges did not seem specific to staff role, one member of domiciliary staff pledging to ‘give a resident a duster’ to encourage resident citizenship by facilitating their sense of purpose and role within the care home community. This pledge could be seen as simplistic but was firmly rooted in that staff member’s clear understanding of the resident’s biography (personhood) and how crucial this was to their wellbeing. Only two pledges did not reflect either personhood or citizenship and two participants declined to complete a pledge.

#### Types and quality of interactions

During baseline and follow-up DCM™ observations, there were a possible 960 five-min time frames for which interactions could be recorded. The majority of interactions (*n*=792) recorded during observation were ‘no interaction’, and this reflected 82.5% of all observed interactions at baseline. At baseline, the proportion of enhancing interactions was balanced between the PERSONABLE (*n*=165) and training as usual (*n*=152) arms; however, at follow-up, the PERSONABLE (*n*=177) arm had a much greater amount of enhancing interactions recorded compared to the training as usual (*n*=108) arm.

The brief qualitative DCM™ notes for each interaction indicated a large proportion of interactions were ‘neutral’ in quality (Table 4). These types of interactions were not overtly detracting and therefore considered enhancing. Examples of these types of interactions were captured in the brief DCM™ field notes ‘brought food, nothing exceptional’, ‘offered food, nothing exceptional’, ‘helped to mobilise, nothing exceptional’, ‘acknowledged, nothing exceptional’ or ‘offered drink, nothing exceptional’.

**Table 4:** Number and proportion of interactions occurring at baseline and follow-up: total number of 5-min observation intervals where an interaction could occur was 960

Time	Interaction	PERSONABLE	Training as usual
Baseline, *n* (%)	Enhancing	165 (17.2)	152 (15.8)
	No interaction	792 (82.5)	806 (84.0)
	Detracting	3 (0.30)	2 (0.20)
Follow-up, *n* (%)	Enhancing	177 (18.3)	108 (11.2)
	No interaction	776 (81.0)	835 (87.0)
	Detracting	7 (0.70)	17 (1.80)

#### Interrater agreement

This study attempted to explore the agreement of two independent DCM™ observers when they had not previously undertaken observations together. The two observers were unfamiliar with each other’s DCM™ practice and had not conferred about the measure prior to conducting observations. The two observers JC and JF conducted 4 h of interrater agreement in one intervention and one training as a usual care home. Both JC and JF observed the same sample of residents (*n*=10) across the intervention and usual care homes. For the behaviour category codes, observers only agreed for 63 out of 339 time frames, a very low concordance coefficient of 18.6%. The two observers had better, but still poor, agreement for Mood and Engagement scores, 188 out of 339 time frames, a low concordant co-efficient of 55.5%.

## Discussion

### Recruitment and retention of participants

There was a positive response from care homes, staff and residents approached for inclusion in this study despite the difficulty in care home recruitment reported by similar studies [5, 26]. Receptivity to inclusion in the study did not seem to be affected by Care Quality Commission ratings, with those rated as ‘good’ or ‘requires improvement’ both responding well to approaches for inclusion. Those care homes rated as ‘outstanding’ were less receptive; however, they were more likely to already have person-centred training in place [14, 25] and therefore does not perceive the intervention as beneficial. Care homes with changes in leadership, between baseline and follow-up, also had high attrition of staff, mainly through leaving employment. Comparatively, a care home where the owner was integrated into the care routines, for example taking residents swimming, retained all staff throughout the study period except for one retirement.

#### Intervention

The level of staff turnover did not necessarily translate into intervention attendance. For example, the care home with the least stability of staff and management had the highest attendance at PERSONABLE of all three intervention sites. In this care home, a senior carer was temporarily acting in a managerial position and worked closely with the administrator in coordinating attendance at PERSONABLE. Despite the brevity of the intervention, care home managers perceived PERSONABLE to be burdensome and had a general reluctance to run the workshop more than once. This limited how much the intervention could integrate the principles of personhood and citizenship into the working culture of a care home. Some conversations about the intervention between attending and non-attending staff were noted during DCM™ observation.

Despite the demands of busy care environments and PERSONABLE being offered just once at each intervention site, staff were highly receptive to the intervention and there was good attendance from participating staff. Of the 44 staff randomised to receive the intervention, 29 undertook the training. Attendance was enhanced when administrative staff or a designated care worker was engaged in the promotion of PERSONABLE. When a manager promoted the workshop in the weeks leading up to PERSONABLE, attendance was better; however, in this instance, a greater proportion of staff attended on their days off. Reasons expressed by staff for attendance on days off were a sense of duty, a willingness to learn, interest in research, an assumption that this was part of their job and perceived pressure from their manager. However, we are mindful that future research funds should either provide for travel expenses for staff attending training on their days off, or this practice should be discouraged at the point of care home recruitment. The pledges constructed within PERSONABLE were aimed at helping staff to take ownership of the PERSONABLE intervention, potentially enabling them to translate their understanding of personhood and citizenship into care provision. Evidence of pledge enactment was observed during follow-up on an individual (personhood) level; however, larger changes to the care community (citizenship) were less apparent.

### Outcome measurements

DCM™ was designed as a practice development tool to describe the resident experience and interactions with staff to prompt reflective discussion. It is an approach for which there is substantial evidence of positive impacts [3]. When DCM™ is used as an outcome measure rather than an intervention, the reflective feedback element is omitted. We found that having too many DCM™ behaviour category codes increased the opportunity for observer disagreement - JC and the second observer frequently referred to the DCM™ handbook but even after discussion would sometimes disagree on the behaviour category code. This suggests the need to ensure observers have adequate time to discuss their interpretation of DCM™ guidance prior to conducting observations, so that any misunderstandings, or variations from usual practice can be addressed. The mood and engagement score being combined dilutes the utility of these distinct concepts; this duality was observed on many occasions, for example, one gentleman was very engaged in a jigsaw throughout baseline and follow-up measurements, despite his facial expression being solemn throughout he was given the higher engagement value as per DCM™ guidance. The adopted binary approach in this study to describe interactions between staff and residents helped to efficiently capture the many interactions. In the context of developing DCM™ as an outcome measure introducing a neutral code would help capture the more benign interactions.

Staff participants reported that the Personhood in Dementia Questionnaire was quick to complete and understand. Only two questionnaires had notes in the margins, these notes were intended to add clarification when a question had been perceived by the participant as ambiguous. Seven staff who attended PERSONABLE did not have sufficient time to both attend the workshop and complete a questionnaire, and this is a consideration for the design of a future study. Staff reported finding the VAS easy to complete; many ancillary and administrative staff reported that they did not feel their work was ‘care’ and sought clarification that the question applied to them. The TESS-RC effectively reflected the impression of the observer of the general quality of the care home; however, the ethical committee requirement that resident bedrooms and other personal spaces be omitted from this measurement may have impacted the overall impression of the care home and subsequent validity and reliability of this measurement tool. Subsequent studies would benefit from developing a tool that captures the training that is delivered within each care home. This would enable researchers to be clearer about differences between trial arms due to the delivered intervention.

## Conclusion

Future trials would benefit from simplification of recruitment strategies, interventions and outcome measurements. Trials can be sympathetic to the demands of the residential care environment by balancing the need to collect sufficient information to make meaningful conclusions and the potential overburdening of study participants. The sheer size of the care home population suggests that thoughtful and energetic efforts to overcome the challenges of research in this complex environment can yield high-powered studies that will ultimately improve the care of residents. Using a flexible approach that does not compromise methodological rigour may improve the recruitment and retention of participants. Delivered interventions do not necessarily need to be lengthy or extensive. In phase one of this study, staff were less enthusiastic about the engagement with, and use of, interventions when they did not fit into their busy work routine. The parts of this study which did not run to plan were those which were not intuitive for participants: the need for blinding, the consultee process and the understanding of staff that there is a need for both baseline and follow-up measurements. A greater research presence and higher visibility of researchers in all participating care homes may help to familiarise residents, owners, managers, staff and family members with the steps necessary to undertake successful trials. A citizenship approach to conducting care home trials may be of benefit. Building a care home research ‘community’ could improve the overall acceptability of research methods to care homes, staff and in particular the resident citizens.

## Data Availability

Data set available on request from the first author.
